# Role of ectonucleotide pyrophosphatase/phosphodiesterase 2 in the midline axis formation of zebrafish

**DOI:** 10.1038/srep37678

**Published:** 2016-11-24

**Authors:** Frisca Frisca, Daniel Colquhoun, Yona Goldshmit, Minna-Liisa Änkö, Alice Pébay, Jan Kaslin

**Affiliations:** 1 Australian Regenerative Medicine Institute, Building 75, Monash University, Australia; 2 Centre for Eye Research Australia, Royal Victorian Eye and Ear Hospital & Ophthalmology, the University of Melbourne, Department of Surgery, Australia; 3 Department of Neurobiology, Tel-Aviv University, Israel; 4 Monash Biomedicine Discovery Institute Department of Anatomy and Developmental Biology, Biomedicine Discovery Institute, Monash University, Australia

## Abstract

Lysophosphatidic acid (LPA) is a unique bioactive lysophospholipid that induces pleiotropic effects in various cell types and organisms by acting on its specific receptors. LPA is mainly synthetised extracellularly by the ectonucleotide pyrophosphatase/phosphodiesterase 2/autotaxin (enpp2). Altered LPA signalling is associated with embryonic abnormalities, suggesting critical roles for LPA during development. However, the role of LPA signalling during early embryogenesis is not well established. We demonstrate that enpp2/LPA signalling in the early zebrafish embryo results in altered axis and midline formation, defects in left right (L-R) patterning, ciliogenesis of the Kupffer’s vesicle (KV), through the modulation of cell migration during gastrulation in a lpar_1–3_ Rho/ROCK-dependant manner. Overall, this study demonstrates an essential role of enpp2/LPA signalling during early embryogenesis.

The midline is an essential embryonic structure during vertebrate embryogenesis. It provides structural support of vertebrates, and plays a role in tissue patterning and in the establishment of the left right asymmetry (L-R) during internal organogenesis^1^,^2^,^3^. The development of the midline/body axis occurs from the onset of gastrulation. The process is initially marked by mesoderm and endoderm progenitor cell compaction at the dorsal side of the gastrula (dorsal organizer shield) which then undergoes simultaneous morphological changes, cellular intercalation and migration (convergent extension, CE) that is governed by specific signalling pathways to form the body axis^4^,^5^. Failure in this process and the associated signalling pathways alter midline formation and result in developmental abnormalities including impaired elongation of the body length^6^,^7^,^8^, establishment of L-R asymmetry^9^ and tissue patterning^10^. Many key signalling molecules and signalling pathways regulate midline formation during embryogenesis, including Platelet-Derived Growth Factor (PDGF)^11^,^12^, the non-canonical Wnt/Planar Cell Polarity (PCP) pathway^13^, Bone Morphogenetic Protein (BMPs)^14^ and Fibroblast Growth Factor (FGFs)^15^. However, the role of phospholipid signalling in this context remains poorly understood.

Lysophosphatidic acid (LPA) is a unique bioactive lysophospholipid that induces pleiotropic effects through binding to its specific G-protein coupled receptors lpa_1–6_ (reviewed in refs 16 and 17) in various cell types. LPA signalling affects proliferation, cell survival, motility, morphological rearrangements and differentiation. Dysregulation of LPA signalling is associated with early and late embryonic abnormalities, suggesting critical roles for LPA during development^18^,^19^,^20^,^21^. Studies in LPA receptor knockout mouse models have shown a prominent role of LPA signalling in particular in neural and vascular development^19^,^22^. Lpa_1_^(−/−)^ mice die postnatally due to an impaired suckling behavior likely associated with a defective olfaction and impaired CNS development^19^. Lpa_2_^(−/−)^ mice do not display severe phenotypes although some intracellular signalling pathways such as PLC activation, Ca^2+^ mobilization, and stress fiber formation are altered^23^. Lpa_3_^(−/−)^ female mice display a reproductive impairment, with delayed embryo implantation and embryo spacing alterations^24^ while Lpa_4_^(−/−)^ mice have defects in blood vessel formation leading to haemorrhages in many organs at different embryonic stages^22^. Lpa_5_^(−/−)^ mice show deficit in response to neuropathic pain (reviewed in ref. 25) and the loss of lpa_6_ in *Xenopus* embryo disrupts forebrain development^26^.

The ectonucleotide pyrophosphatase/phosphodiesterase 2 (enpp2), also known as autotaxin (atx) or lysophospholipase D, is the main enzyme responsible for the synthesis of extracellular LPA^27^,^28^. The loss of enpp2 in mice is lethal between E9.5–E10.5 because of severe vascular defects in the yolk sac and embryo^25^,^29^,^30^, hampering the characterization of the LPA function in the early mouse embryo. In the early zebrafish embryo, the *enpp2*/*lpa*_*3*_ axis regulates the L-R internal organ asymmetry but the molecular mechanisms responsible are unknown^31^. Furthermore, overexpression of *enpp2* in zebrafish induces cardia bifida^32^. Enpp2/LPA signalling also regulates oligodendrocyte production and differentiation of the zebrafish hindbrain^33^. Although these studies suggest essential roles for LPA signalling during embryogenesis, the understanding of LPA functions remains limited.

To determine the function of LPA during embryogenesis, we transiently overexpressed the LPA-producing enzyme enpp2 in developing zebrafish. Our gain of function study of *enpp2* in the zebrafish embryo shows for the first time the unique role of *enpp2* in regulating the cell migration during gastrulation and subsequent formation of the axial midline, as well as the establishment of L-R asymmetry. Furthermore, rescue experiments show that these effects are lpa_1–3_-dependent and mediated by the Rho/ROCK pathway.

## Results

### Overexpression of enpp2 alters axis formation in the zebrafish embryo and modulates expression of the midline axis genes *shha* and *ntl*

**Figure 1:**
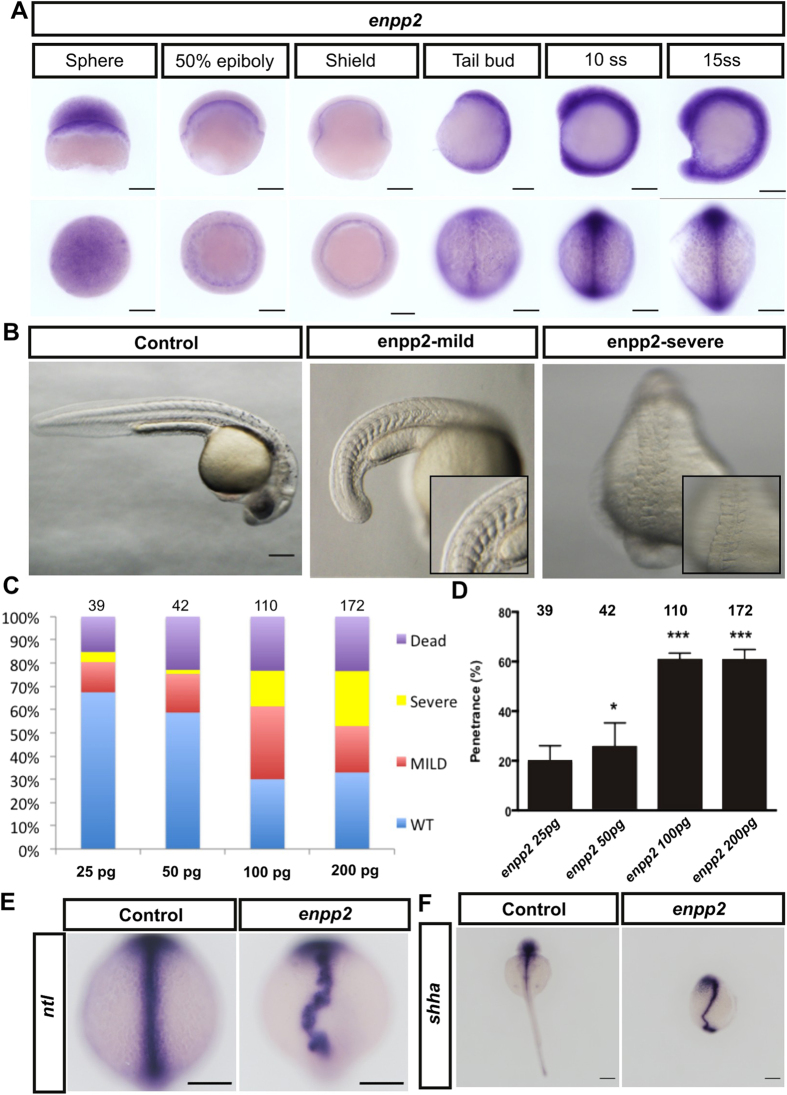
Enpp2 overexpression alters midline axis formation in the early embryogenesis of zebrafish. Developmental series of WISH at designated stages were performed using *enpp2* antisense riboprobe. (**A**) Lateral view and animal/dorsal view of WISH early zebrafish embryos at designated stages. (**B**) Representative pictures of control, mild (slight delay and smooth somite borders) and severe (developmental delay and midline axial defect) phenotypes in *enpp2* injected embryos. (**C**,**D**) Quantification of phenotype variant and penetrance (detectable phenotype) following injection with different doses of enpp2 RNA and normalized to control. The sample size (n) is stated as numerical value above each bar. Data are mean ± SEM from at least four independent experiments. Statistical analysis was established by one-way ANOVA; *P < 0.05; ***P < 0.001. Dead embryos were normalised to uninjected embryos (**E**) Representative WISH pictures of control and enpp2 injected embryos with midline axis gene probes *ntl* or *shha* riboprobes (**F**). >50 embryos used in each experiment. Scale bars: 200 μm.

We cloned the full-length *enpp2* (NM_200603.1)^34^ to generate probes for whole mount *in situ* hybridization (WISH). WISH analysis of the developing zebrafish embryos showed that *enpp2* mRNA expression is dynamically regulated during development, which was also confirmed by quantitative RT-PCR analysis (Fig. 1A, Suppl. Fig. 1). *Enpp2* was maternally deposited (sphere) and expressed at a very low level in the yolk syncytial layer (YSL) during the early gastrulation stage (50% epiboly and shield) (Fig. 1A, Suppl. Fig. 1A,B). At the end of gastrulation (tail bud), *enpp2* was notably expressed in the midline axis and its levels continued to increase during the segmentation period (10 somite stage, 15 somite stage) (Fig. 1A, Suppl. Fig. 1), suggesting a potential role in midline and axis formation. In order to assess the role of enpp2 during embryogenesis, we injected increasing amounts of capped *enpp2* mRNA into the zebrafish embryo (1–4 cell stage), determined the morphology (Fig. 1B) and measured the phenotype penetrance (Fig. 1C). The overexpression of enpp2 resulted in significant axis defects and in a kinked notochord in a dose-dependent manner (Fig. 1B–F). This was accompanied by aberrant somite shapes, highlighted by the lack of chevron-shaped somites and shortened body length (Fig. 1B). Embryos injected with *enpp2* mRNA at increasing concentrations exhibited dose-dependent penetrance of phenotypes (25 pg: 19.3 ± 6.8%; 50 pg: 25.6 ± 9.6%; 100 pg: 60.7 ± 2.7%; 200 pg: 60.5 ± 4.3%, Fig. 1D). These phenotypes suggest midline and axis defects during embryogenesis. To examine this further, we performed WISH to assess the expression patterns of midline markers *shha* and *ntl* to mark the notochord of the zebrafish embryo at 10 somite stage and 24 hours post fertilization, respectively (Fig. 1E,F). The majority of enpp2 -overexpressing embryos displayed abnormal pattern of *shha* and *ntl* expression, characterized by kinked, patchy or expanded patterns, or expression in multiple buds (duplicated), which indicated a notochord defect. This finding suggests a role of enpp2 in regulating the midline formation and its impact on the expression of the midline axis genes *shha* and *ntl*.

### Enpp2 overexpression induces L-R patterning defect

**Figure 2:**
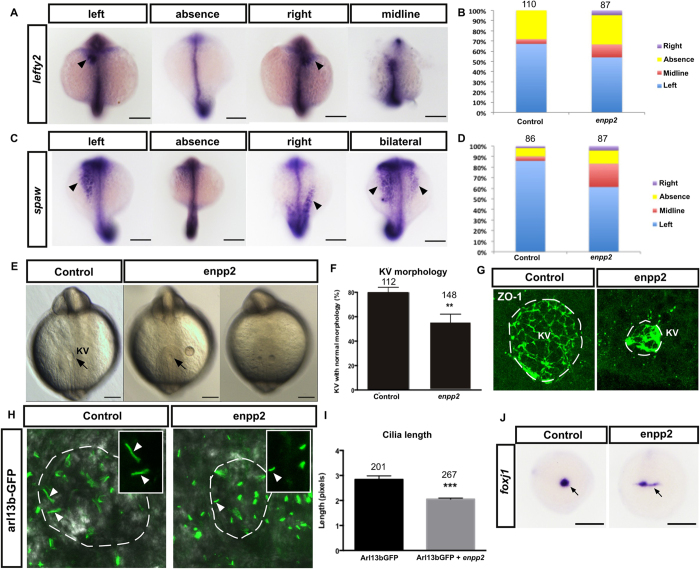
Enpp2 modulates nodal-related asymmetry gene expressions, KV formation and ciliogenesis. Representative images of WISH of the control and enpp2-injected zebrafish embryos at 15–21ss with *lefty2* (**A**) and *spaw* (**C**) antisense riboprobes respectively. The midline/notochord is highlighted by *ntl* expression. The determination of left, bilateral, absence, and right groups was based on the location of *lefty2* or spaw expression compared to *ntl* (midline). The quantification of *lefty2* and *spaw* expression distribution embryos is shown in (**B**) and (**D**) respectively. (**B**) The proportion of *lefty2* expression on the left, bilateral, absence and right side are 67.3%, 4.5%, 28.2%, and 0% respectively in the control embryos and 54%, 12.6%, 28.7%, and 4.6% respectively in the enpp2 overexpressed embryos. (**D**) The proportion of *spaw* expression on the left, bilateral, absence and right side are 86%, 4%, 8%, and 2% respectively in the control embryos and 61.3%, 21.8%, 12.7%, 4.2% respectively in the enpp2-overexpressed embryos. (**E**) Representative images of the KV morphology in control- and in enpp2 overexpressing embryos (5–8ss). (**F**) Quantification KV size in enpp2 overexpressed embryos compared to control. (**G**) Whole-mount antibody staining of zona occludens 1 (ZO-1) that labels cell junctions and outlines the KV in control enpp2 injected embryos. (**H**) Representative confocal Z-stack images of cilia in the KV, marked with arl13b GFP, highlighting cilia morphology and distribution in control- and *enpp2* overexpressing embryos at 5–8ss. (**I**) Quantification of the cilia length between control- and enpp2 overexpressing embryos. The sample size (n) is stated as numerical value above each bar. Data are mean ± SEM Statistical analysis was established by t-test; ***P < 0.001. n indicated above bars. White circles highlight the KV lumen. (**J**) Enpp2 overexpression altered the expression pattern of *foxj1*, a master regulator of ciliogenesis, observed at 90% epiboly embryo.

Establishment of the midline is important for L-R asymmetry and loss of enpp2/LPA signaling has been linked to asymmetry phenotypes^31^,^35^,^36^,^37^. To establish if increased enpp2/LPA signaling alters the L-R patterning of the zebrafish embryo, we examined the expression of the nodal-related asymmetric genes, *lefty1/2* and *southpaw* (*spaw*) in the lateral plate mesoderm (LPM) during somite stages in control wild type and *enpp2*-overexpressing zebrafish. As shown in Fig. 2A–D, *lefty1/2* and *spaw* were mainly expressed on the left side of the LPM during mid-late somitogenesis (15–21ss) in the control wild type zebrafish, which is consistent with previous reports^38^,^39^. However, following *enpp2* overexpression, the zebrafish displayed a more randomized expression pattern of these genes, either right-sided, absent, or bilaterally expressed in the LPM compared to control (Fig. 2A–D). In addition, the *spaw* expression domain was shifted posteriorly in some of the *enpp2* overexpression embryos suggesting that migration of the lateral plate mesoderm is delayed or perturbed (Fig. 2A–D). Taken together, this demonstrated that *enpp2* overexpression modulates L-R patterning and maintenance of the expression of the nodal-related asymmetric genes *lefty1/2* and *spaw*.

### Enpp2 modulates the organogenesis and ciliogenesis of the KV

In the zebrafish, the Kupffer’s vesicle (KV) plays an important role in the establishment of L-R patterning by maintaining the flow of nodal morphogens along the body axis^40^. Ciliated cells in the KV drive gradients of signalling molecules that shape the L-R asymmetry. We next investigated if the formation and morphology of the KV were altered by enpp2 overexpression. Firstly, we examined the lumen shape of the KV at the 6–8-somite stage, when the KV is formed and nodal morphogen flow occurs^40^. In control embryos, the KV appeared as a flattened sphere with a single round lumen, while in the overexpressed-*enpp2* fish, the lumen was smaller, misshapen or absent (Fig. 2E–G). Quantification of KV size showed that the lumen size was dramatically reduced in the enpp2 overexpressed embryos (45.00 ± 7.1%) compared to control (18.46 ± 6.80%) (Fig. 2F). To examine KV cilia, we performed live imaging using the axonemal marker arl13b-GFP^41^. Live imaging showed that the cilia lengths in the KV were significantly reduced following enpp2 overexpression (2.00 ± 0.04 units), compared to control (2.80 ± 1.44, p < 0.001, n > 200, Fig. 2H,I). This data indicates that enpp2 regulates cilia formation. *Foxj1* is a master regulator of ciliogenesis and labels cells in the KV^42^,^43^. Analysis of *foxj1* expression in enpp2 overexpressing embryos showed de-clustered pattern of *foxj1* expression pattern (Fig. 2J). In control embryos *foxj1* expression was clustered and compacted in an ovoid-like shape; in enpp2 -overexpressing embryos *foxj1* was significantly de-clustered to a linear domain (arrow, Fig. 2J).

### Enpp2 overexpression induces defects in the midline axis and in the KV organogenesis

**Figure 3:**
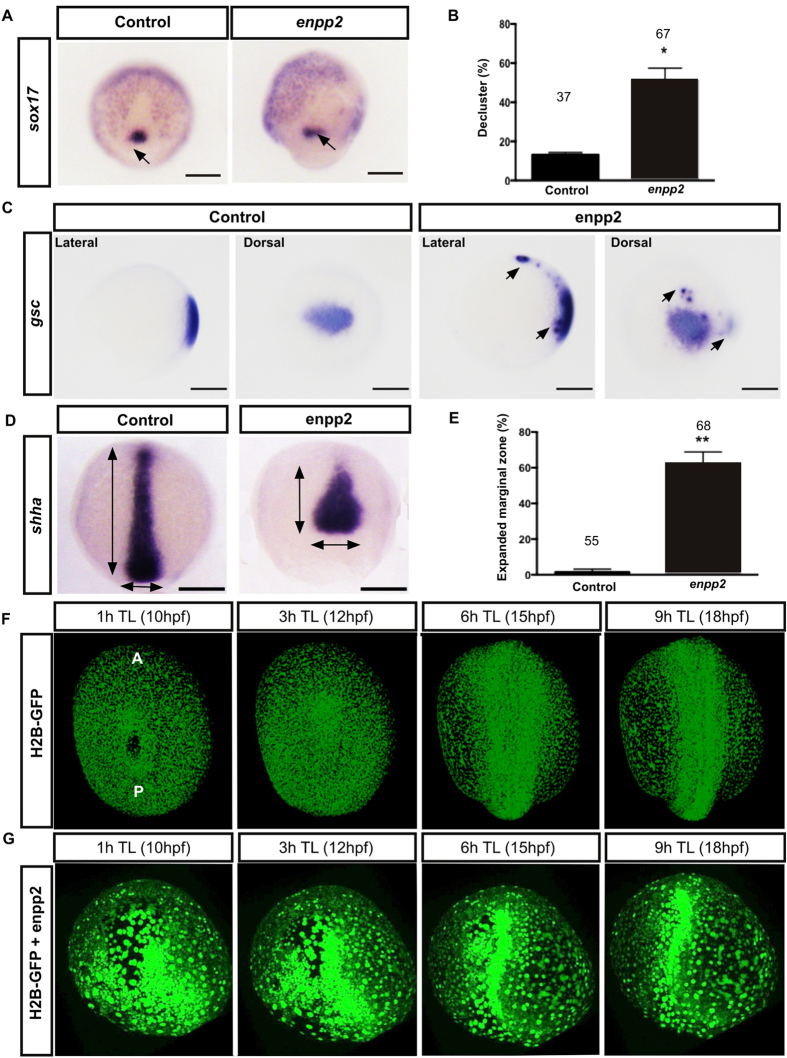
Enpp2 overexpression induces alteration in cellular migration, the expression pattern of KV precursors, dorsal organizer and midline genes (*gsc* and *shha*) during early embryogenesis. (**A**) Representative images of WISH with *sox17* riboprobe to mark the integrity of the DFC cluster at 75% epiboly. (**B**) Quantification of KV defect and/alteration of DFC cluster, as highlighted by *sox17* WISH. (**C**) The *gsc* expression is observed in the dorsal organizer of enpp2 overexpressing embryos. However, the expression is also detected in the axial mesoderm precursors suggesting that convergence and cell migration during midline formation is impaired. (**D**) Representative images of WISH of *shha* at 90% epiboly showing a truncated axis and laterally expanded expression of shha at the blastomderm margin. (**E**) Quantification of the broadened shha expression domain at the blastoderm margin in control and enpp2 overexpressing embryos at 90% epiboly. (**F**,**G**) Representative images of time lapse series showing cell migration during midline formation (10 hpf- early somitogenesis) in wild type and *enpp2*-injected embryos. Image stacks were taken every 15 minutes. Time course for each image is indicated. The enpp2 overexpressed embryo failed to undergo a proper CE (**G**) which results in bend and broadened midline compared to wild type (**F**). (**B,E**) The sample size (n) is stated as numerical value above each bar. Data are mean ± SEM. Statistical analysis was established by t-test; *P < 0.05; **P < 0.01. n indicated above bars.

The KV formation is dependent on the correct migration and clustering of distinct precursor cells, the dorsal fore runner cells (DFC), during mid-gastrulation (75% epiboly stage)^44^,^45^. The DFC express *sox17* during early embryogenesis^45^. In enpp2 overexpressing embryos, the expression of *sox17* is readily observed, indicating that the specification of the DFC is not altered. However, the cluster formation of the DFC is significantly impaired in enpp2 overexpressing embryos (51.2 ± 6.2%) compared to control (13.1 ± 1.1%). Enpp2 overexpressing displayed an altered pattern of *sox17 expression* (Fig. 3A,B), similar to the one observed with *foxj1* expression (Fig. 2F). The disorganisation of the DFC could lead to reduced or absent of KV formation. By measuring and observing the KV at stage 6–8ss, we detected a smaller or absent KV in *enpp2* overexpressing embryos (Fig. 2E). The de-clustered expression of *sox17* and *foxj1* expressions suggest the observed phenotypes could be caused by the impaired cell migration during gastrulation.

Since we found the convergence and extension movements (CE) during midline formation were impaired, we next assessed if the dorsal organizer is formed correctly following enpp2 overexpression by performing WISH using *goosecoid* (*gsc*), a dorsal organizer marker^46^, during the mid epiboly stage (Fig. 3C). We observed *gsc* expression in the dorsal organizer but *gsc* expression was also detected in the axial mesoderm, suggesting that precursors do not properly converge into the dorsal organizer during midline formation (Fig. 3C). Consistent with this result, the expression of the axial mesoderm marker *shha* showed expanded expression at the blastoderm margin and a shortened anterior to posterior expression pattern in enpp2 injected embryos (61.96 ± 6.8% in the enpp2 overexpressed embryo compared to 1.58 ± 1.6% in control, Fig. 3D,E) confirming an impaired convergence extension during gastrulation. We next performed time-lapse analysis of cell movement from the end of gastrulation until early somitogenesis to observe the midline formation. Cell migration defects were detected during epiboly in enpp2 overexpressing embryos (Fig. 3F,G, Suppl. Movie 1–2). In wild type embryos, cells intercalated and initiated the elongation during mid-line formation, the cells in the enpp2 overexpressing embryos failed to converge and extend, resulting in a broader midline area and a bent midline formation (Fig. 3F,G, Suppl. Movie 1–2). The detected cell migration phenotypes were in agreement with the altered gene expression patterns of *gsc, ntl, spaw* and *shha* in enpp2 overexpressing embryos.

### *enpp2* modulates cell migration via lpar_1–3_ Rho/ROCK

In order to dissect the signalling mechanisms underlying LPA’s effect in development, we first examined the expression profile of its receptors (lpa_1–3_) in the early embryo by WISH. We detected expression of lpa_1–3_ in the early embryo (Suppl. Fig. 2 and data not shown). We found that lpa_1–3_ were ubiquitously expressed at the animal portion and at the margin between cells and yolk portion (yolk syncytial layer) during blastula until onset of gastrulation. During the late gastrulation and early segmentation stages, *lpa*_*1*_ was expressed in the mesoderm tissue adjacent to the body axis, while *lpa*_*2–3*_ were expressed along the midline during zebrafish (Suppl. Fig. 2). Taken together, the expression of *lpa*_*1*–*3*_ suggests that these receptors may play an important role during gastrulation and the midline formation.

**Figure 4:**
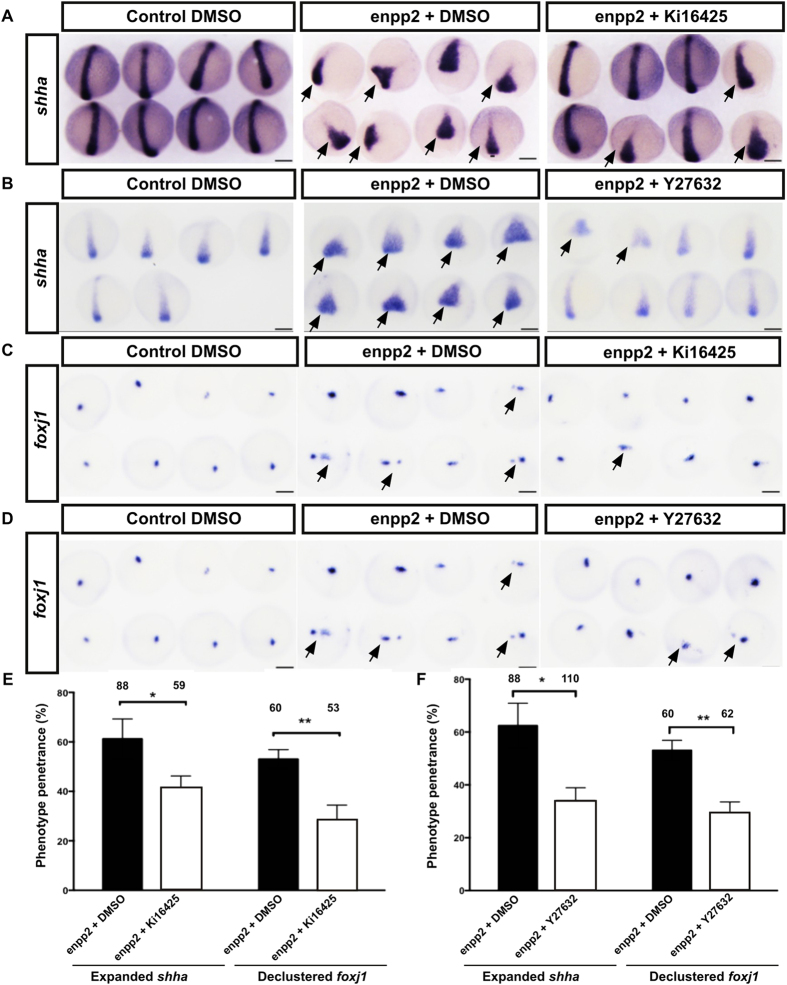
Pharmacological blocking of lpa_1–3_ or Rho/ROCK signalling rescue enpp2 overexpression induced midline phenotype. Representative WISH images of *shha* (**A,B**) and *foxj1* expression (**C-D**) at 90% epiboly of enpp2 overexpressing embryos treated with the lpa_1–3_ antagonist Ki16425(**A**,**C**) and Rho/ROCK signalling inhibitor Y27632 (**B**,**D**). Enpp2 overexpressing embryos display expanded expression of *shha* (**A**,**B**) and *foxj1* (**C**,**D**) illustrated by pointed in arrows. Quantifications of the of shha expression domain in enpp2 overexpressing embryo following vehicle or Ki16425 (**E**) or Y27632 treatment (**F**). The phenotype penetrance following enpp2 injection and Ki16425 treatment and Y27632 were measured at 90% epiboly (**E**,**F**). (**E**,**F**) The sample size (n) is stated as numerical value above each bar from at least two independent experiments. Data are mean ± SEM. Statistical analysis was established by t-test; *P < 0.05; **P < 0.01. n indicated above bars.

We next addressed the role played by LPA receptors in the phenotypes observed after enpp2-overexpression. To this end we performed rescue experiments using the well-established LPA receptor (Lpa_1–3_) antagonist Ki16425^47^. Ki16425 is a specific lpa receptor antagonist that efficiently antagonize lpa_1–3_ but not lpa_4_, lpa_6a_, and lpa_6b_ and it has been used to antagonise LPA signalling in zebrafish previously^34^. We incubated enpp2-overexpressing embryos immediately after RNA injection with increasing concentrations of Ki16425 and assessed the impact on phenotypes by using *shha* and *foxj1* expression as a read-out at the end of gastrulation (Fig. 4). *enpp2* injected embryos treated with Ki16425 showed a significant reduction of ennp2-induced CE and ciliogenesis phenotypes in a dose-dependent manner (Fig. 4A–C). Following *enpp2* overexpression, the CE phenotype characterised by a short axis and a broadened *shha* expression was detected in 66.5 ± 7.8% of the injected embryos (Fig. 4E). However, following and Ki16425 treatment, the number of embryos displaying this phenotype decreased to 41.3 ± 4.9% (Fig. 4E). Similarly, declustered *foxj1* expression was observed in 53 ± 3.8% of the vehicle control embryos but decreased to 28.3 ± 6.2% in Ki16425-treated embryos (Fig. 4E). The rescue effect was maintained until later stages of development as we observed significantly reduced morphological phenotypes and almost normal expression levels of *ntl, shha* and its downstream gene *gli2* following Ki16425 treatments at 24 hours post fertilisation (hpf, Fig. 2, Suppl. Fig. 3).

Rho/ROCK is one of the common downstream pathways of enpp2/LPA signalling and it plays an essential role in regulating cell shape and migration through actin stabilization of the cytoskeleton^16^,^48^. Furthermore, the Rho/ROCK pathway is an important regulator of CE cell movement during midline formation^49^,^50^. We inhibited the Rho/ROCK pathway by using a selective inhibitor of the Rho-associated protein kinase p160 ROCK, Y27632^51^. Inhibiting Rho/ROCK after enpp2 overexpression significantly reversed the observed phenotypes. The penetrance and severity of CE and clustering of DFC were significantly reduced after Y27632 treatment (Fig. 4B,D,F). Y27632 treatment reduced the expanded *shh* expression phenotype from 62.4 ± 8.5% in the vehicle control to 33.9 ± 4.9% in treated embryos (Fig. 4F). The *foxj1* expression pattern was 53 ± 3.8% in the vehicle control and decreased to 29.2 ± 4.3% in Y27632-treated embryos (Fig. 4F). The rescue effect of Y27632 in enpp2 injected embryos was maintained until later stages of development as we observed significantly reduced morphological phenotypes and almost normal expression patterns of *ntl* and *spaw* at 24 hpf (Suppl. Fig. 3). Taken together this data indicates that enpp2 induces the midline axis phenotype through activation of the Rho/ROCK pathway in a lpa_1–3_ mediated manner.

## Discussion

The role of LPA signalling in early embryonic development is limited^19^,^22^,^23^,^24^,^31^. Here we found a role of enpp2/LPA signalling in cell migration during gastrulation, which interfered with the midline axis formation and the establishment of the L-R asymmetry.

The establishment of the axial/midline mesoderm and the L-R asymmetry are crucial to the formation of the body plan in the zebrafish embryo. The body axis arises from the dorsal organizer shield^6^,^52^,^53^. Midline formation involves a complex morphogenetic movement, called the convergence and extension movement. During this process, the axial mesoderm cells migrate together and intercalate (converge) towards the dorsal organizer and then migrate towards the anterior and posterior poles (elongation) to give rise to the future midline^4^,^5^,^54^. *Enpp2* is expressed at the blastula stage in the zebrafish and it is highly expressed in the margin and midline during gastrulation and somitogenesis respectively. During epiboly, *enpp2* is expressed in the YSL and it is later expressed in the dorsal organizer suggesting a role in tissue patterning. *lpa*_*1*–*3*_ are also expressed in the embryo during gastrulation. During early gastrulation *lpa*_*1*_ is expressed at the dorsal organizer shield, where the CE occurs to form the body axis^54^. At the end of gastrulation *lpa*_*1*_ expression is found in the mesoderm adjacent to the midline. In contrast*, lpa*_*2b*–*3*_ are expressed at the midline and at the end of gastrulation. These expression patterns suggest that enpp2/LPA signalling is involved in epiboly and gastrulation. To assess the role of enpp2 in the zebrafish embryogenesis, we overexpressed *enpp2* mRNA and assessed the embryonic phenotype at different stages of the zebrafish development. We observed an alteration of cell migration towards the midline during gastrulation. Cells failed to complete the CE *e.g*. to migrate towards the dorsal organizers and to elongate to form the midline, resulting in perturbed expression of midline and lateral plate mesoderm genes (*shha ntl* and *spaw*), bent body axis and shortened body length. The impaired cell migration during CE is likely responsible for the expanded expression of midline genes, bend/kinked midline in the enpp2 -overexpressing embryo and the shortened body plan at later stages of development. The Rho/ROCK pathway is a common and a likely downstream pathway of the enpp2/LPA signalling axis, and particularly through multiple LPA G-protein coupled receptors, including the lpa_1–6_^16^,^48^. Rho/ROCK is a key regulator of cell shape and migration through its action on actin stabilization of cytoskeleton^49^. Rho/ROCK pathway is an important regulator of CE process during midline formation and regulates left-right asymmetry^49^,^50^. Indeed ROCK has been previously shown to play an obligatory role in the morphogenetic movement during embryonic organogenesis. Inhibiting ROCK with Y27632 in the early chick embryo blocked cell migration and fusion of the bilateral heart primordia, axis formation, movement of Hensen’s node (similar to KV), and establishment of L-R asymmetry^50^. We demonstrated that the enpp2 overexpression midline defect phenotype could be rescued by blocking ROCK, suggesting that the phenotype is mediated by Rho/ROCK. In agreement with our findings, the Rho/ROCK pathway regulates CE cell movement during gastrulation^49^,^55^. *rock2b* in zebrafish is specifically expressed in DFC and KV and later in midline^56^. Knock-down of *rock2b* function using morpholinos leads to altered anterior posterior patterning, random distribution of ciliated cells and loss of L-R symmetry in the KV^56^. Similarly, loss of Rock2 function in mice alters organ laterality and asymmetry gene expression including the heart, hypochord and notochord^50^. Interestingly, over activation of sphingosine-1-phosphate (S1P) signalling via its cognate G protein-coupled receptor S1pr2 results in a very similar endoderm convergence phenotype as enpp2^57^. This phenotype is mediated via a RhoGEF-dependent pathway^57^ and implies shared downstream mechanisms. Rho/ROCK is also downstream of the planar cell polarity (PCP) pathway that is a well-known regulator of CE during gastrulation. The vertebrate PCP pathway is a non-canonical Wnt signalling cascade that can modulate the actin cytoskeleton via small GTPases including Rho^58^ to control polarized cell behaviours such as CE movements and orientation of stereocilia in the inner ear^49^,^59^,^60^,^61^,^62^. An intriguing possibility is that LPA signalling interacts with the PCP pathway via the extracellular matrix. Enpp2 has several positively charged surface residues and it may interact with heparin sulphate proteoglycans like glypicans that are well known regulators of the PCP pathway^63^,^64^.

Furthermore, overexpression of *enpp2* altered the migration and clustering of dorsal fore runner cells. This leads to perturbed formation of the KV and ciliogenesis, and subsequently to a failure to establish a correct L-R asymmetry in the embryo. Overexpression of *enpp2* could result in effects independent of LPA signalling. However, the efficient pharmacological rescue with the specific LPA receptor (Lpa_1–3_) antagonist Ki16425 suggests that the phenotypes observed are mediated by LPA receptors. Furthermore, our findings are consistent with the previously reported roles of enpp2/LPA signalling during L-R patterning in mice and zebrafish. Loss of enpp2 in mice leads to phenotypes such as: axial mesoderm/notochord defects, and abnormal organ positioning^29^,^65^, and are associated with abnormal L-R patterning^66^ Furthermore, knock-down of enpp2 or lpa_3_ in zebrafish using morpholinos results in altered KV formation, ciliogenesis and L-R asymmetry^31^. In addition, over activation of *enpp2*/LPA signalling induces cardiac bifida in zebrafish^32^. Cardiac bifida is caused by the failure of bilateral mesoderm to migrate and coalesce into a single central heart tube^67^. Similar to *enpp2*/LPA signaling, over activation of sphingosine-1-phosphate (S1P) signalling via its cognate G protein-coupled receptor S1pr2 impairs endoderm convergence and results in cardia bifida^57^. Taken together, available data suggests that LPA signaling needs to be tightly regulated during multiple stages of embryonic development.

## Conclusion

Midline formation and establishment of L-R symmetry is crucial in the embryonic development. By overexpressing enpp2 in the zebrafish embryo, our study for the first time shows the unique role of enpp2/LPA in regulating mesendoderm cell migration in the establishment of axial midline and L-R asymmetry in the embryogenesis through Rho/ROCK pathway via lpa_1–3_.

## Material and Methods

All experimental work performed in this study was approved by the Monash Animal Ethics Committee (MARP/2013/096), in accordance with the requirements of the National Health & Medical Research Council of Australia.

### Zebrafish

Zebrafish embryos were collected immediately after fertilization, maintained at 28.5 °C, and staged by developmental time (hours post-fertilization, hpf) using morphological criteria^68^. The Tübingen was used as a wild type strain. To perform *in situ*, the embryos at the designated stages were fixed in 4% PFA (2 hrs RT (<24 hpf) or overnight 4 °C)), washed with PBS-0.1% Tween (PBS-T, 5 min, twice), dechorionated, dehydrated with MeOH (5 min RT, twice) and stored in MeOH at −20 °C until processed for *in situ*.

### Microinjection and phenotypic analysis

The embryos were injected with 1 nl of the corresponding RNA at different concentrations. Full-length *enpp2/atx* constructs (25, 50, 75, 100, 150 and 200 pg) or Arl13b GFP mRNA (50–100 pg) or H2B (75 pg) were injected into the 1–4 cell stage embryos. Phenotype penetrances and mortality rates were quantified within 9–24 hrs post injection. For assessment of Kupffer’s vesicle (KV), embryos were examined at the 5–8 somite stages.

### Quantitative Polymerase Chain Reaction (qPCR)

qPCR was carried out using TaqMan Universal master mix (Applied Biosystems, Foster City, CA) and the 7900HT Fast Real-Time PCR system (Applied Biosystems) and SYBR Green (Sigma). Primer set targeting *enpp2*: forward (TTCTCCTTCATCCTTCCACAC) and reverse (GTAACTCCACATCTCGCAGG) (NM_200603) were used. A total of 10 μl reaction was prepared and repeated in triplicate per test. The relative quantitation was achieved by applying the comparative CT method (ΔΔCT) whereby the mRNA levels were normalized against the level of glyceraldehyde-3-phosphate dehydrogenase (GAPDH) mRNA with *enpp2* expression at the shield stage used as the reference.

### Whole Mount *In Situ* Hybridization (WISH)

WISH was performed following the established method with minor modifications^69^,^70^. The primer sequence used for PCR amplification and cloning the following genes, *lpa*_*1*–*3*_, *enpp2/atx, shha, foxj1* (Suppl. Table 1). The resulting DNA fragments were subcloned into pGEMT-easy vector or pCS2+ vector; of which were digested to be transcribed into sense (*enpp2*) and/or antisense mRNA sequence/probe.

### Embryo Immersion/Drug treatment

Following *enpp2/atx* mRNA injection, embryos were immersed in Ringer’s solution containing 0.5% DMSO and supplemented with the Rho/ROCK inhibitor, Y27632 (5 and 10 μM, Sigma-Aldrich) or lpar_1–3_ antagonist, Ki16425 at different concentrations (1, 5, 7.5 and 10 μM Sigma-Aldrich). Y27632 was used at 10 μM and Ki16425 was used at 7.5 μM for the rescue experiments. WT control embryos immersed with the drugs from early gastrulation and embryos did not develop detectable phenotype at 24 hpf. The embryos were then collected and fixed with 4% PFA and processed for WISH.

### Live Imaging

To visualize the KV, wild type or over-expressed zebrafish embryos were dechorionated at 5–8ss stage and mounted in 0.8–1% of Low Melting-point Agar (LMP, Sigma). The KV was then visualized using an Olympus Dissecting microscope MVX10 using the CellSens Standard software (v8.1, Olympus). In some experiments, cilia in embryos KV were visualized under a Leica SP5 confocal microscope and the Leica application suit advanced software (v3, Leica) using a 20 × 1.0 NA water immersion lens. For imaging of cilia, Z-projections of the entire KV were captured with slow acquisition with some frame averaging using *arl13b-GFP,* a live axoneme marker of cilia, to investigate cilia in the living embryo as studied in ref. 41. To image cell migration during midline axis formation, the embryos were injected with H2B together with or without enpp2/atx and visualized starting at the tail bud stage (10 hpf) and recorded by time lapse for 8–10 hours. The embryo was visualized every 10–15 minutes.

### Statistical analysis

All sets of experiments were performed at least three times in triplicates, unless specified (n refers to the number of independent experiments performed on different cell cultures). Data-sets were expressed as mean ± standard error of the mean (SEM). Significance of the differences was evaluated using the unpaired t-test (two-tailed) or the one and two-way ANOVA followed by Dunnett’s multiple comparisons test in the *in vivo* experiment. Statistical significance was established at *p < 0.05, **p < 0.01 and ***p < 0.001, ****=<0.0001. α = 0.5, using Prism (v.6, GraphPad).

## Additional Information

**How to cite this article**: Frisca, F. *et al*. Role of ectonucleotide pyrophosphatase/phosphodiesterase 2 in the midline axis formation of zebrafish. *Sci. Rep.* **6**, 37678; doi: 10.1038/srep37678 (2016).

**Publisher's note:** Springer Nature remains neutral with regard to jurisdictional claims in published maps and institutional affiliations.

## Supplementary Material

Supplementary Figures 1–3

Supplementary Movie 1

Supplementary Movie 2

## References

[b1] Cleaver O., Seufert D. & Krieg P. Endoderm patterning by the notochord: development of the hypochord in Xenopus. Development (Cambridge, England) 127, 869–879 (2000). 10.1242/dev.127.4.869 10648245

[b2] Isogai S., Lawson N. D., Torrealday S., Horiguchi M. & Weinstein B. M. Angiogenic network formation in the developing vertebrate trunk. Development (Cambridge, England) 130, 5281–5290, doi: 10.1242/dev.00733 (2003). 12954720

[b3] Lawson N. D., Vogel A. M. & Weinstein B. M. *sonic hedgehog* and vas*cular endothelial growth factor* Act Upstream of the Notch Pathway during Arterial Endothelial Differentiation. Developmental cell 3, 127–136 (2002). 12110173 10.1016/s1534-5807(02)00198-3

[b4] Tada M. & Heisenberg C.-P. Convergent extension: using collective cell migration and cell intercalation to shape embryos. Development (Cambridge, England) 139, 3897–3904, doi: 10.1242/dev.073007 (2012). 23048180

[b5] Schier A. & Talbot W. Molecular genetics of axis formation in zebrafish. Annu. Rev. Genet. (2005). 10.1146/annurev.genet.37.110801.143752 16285872

[b6] Saúde L., Woolley K., Martin P., Driever W. & Stemple D. Axis-inducing activities and cell fates of the zebrafish organizer. Development (Cambridge, England) 127, 3407–3417 (2000). 10.1242/dev.127.16.3407 10903167

[b7] Odenthal J. . Mutations affecting the formation of the notochord in the zebrafish, Danio rerio. Development (Cambridge, England) 123, 103–115 (1996). 10.1242/dev.123.1.103 9007233

[b8] Talbot W. . A homeobox gene essential for zebrafish notochord development. Nature 378, 150–157, doi: 10.1038/378150a0 (1995). 7477317

[b9] Lenhart K. F., Lin S. Y., Titus T. A., Postlethwait J. H. & Burdine R. D. Two additional midline barriers function with midline lefty1 expression to maintain asymmetric Nodal signaling during left-right axis specification in zebrafish. Development (Cambridge, England) 138, 4405–4410, doi: 10.1242/dev.071092 (2011). PMC3177310 21937597

[b10] Macdonald R. . Midline signalling is required for Pax gene regulation and patterning of the eyes. Development (Cambridge, England) 121, 3267–3278 (1995). 10.1242/dev.121.10.3267 7588061

[b11] Montero J.-A., Kilian B., Chan J., Bayliss P. E. & Heisenberg C.-P. Phosphoinositide 3-kinase is required for process outgrowth and cell polarization of gastrulating mesendodermal cells. Current biology 13, 1279–1289 (2003). 12906787 10.1016/s0960-9822(03)00505-0

[b12] Kai M., Heisenberg C.-P. & Tada M. Sphingosine-1-phosphate receptors regulate individual cell behaviours underlying the directed migration of prechordal plate progenitor cells during zebrafish gastrulation. Development (Cambridge, England) 135, 3043–3051, doi: 10.1242/dev.020396 (2008). 18701549

[b13] Ulrich F., Krieg M., Schötz E., Link V., Castanon I. . Wnt11 functions in gastrulation by controlling cell cohesion through Rab5c and E-cadherin. Developmental cell (2005). 10.1016/j.devcel.2005.08.011 16198297

[b14] Myers D. C., Sepich D. S. & Solnica-Krezel L. Bmp activity gradient regulates convergent extension during zebrafish gastrulation. Developmental biology 243, 81–98 (2002). 11846479 10.1006/dbio.2001.0523

[b15] Fürthauer M., Thisse C. & Thisse B. A role for FGF-8 in the dorsoventral patterning of the zebrafish gastrula. Development (Cambridge, England) 124, 4253–4264 (1997). 10.1242/dev.124.21.4253 9334274

[b16] Frisca F., S. R., Goldshmit Y. & Pébay A. Biological effects of lysophosphatidic acid in the nervous system. Int Rev Cell Mol Biol 296, 273–322 (2012). 22559941 10.1016/B978-0-12-394307-1.00005-9

[b17] Choi J. W. . LPA Receptors: Subtypes and Biological Actions. Annual Review of Pharmacology and Toxicology 50, 157–186, doi: 10.1146/annurev.pharmtox.010909.105753 (2010). 20055701

[b18] Kingsbury M. A., Rehen S. K., Contos J. J., Higgins C. M. & Chun J. Non-proliferative effects of lysophosphatidic acid enhance cortical growth and folding. Nat Neurosci 6, 1292–1299 (2003). 14625558 10.1038/nn1157

[b19] Contos J. J. A., Fukushima N., Weiner J. A., Kaushal D. & Chun J. Requirement for the lpA1 lysophosphatidic acid receptor gene in normal suckling behavior. Proceedings of the National Academy of Sciences of the United States of America 97, 13384–13389 (2000). 11087877 10.1073/pnas.97.24.13384 PMC27233

[b20] Yung Y. C. . Lysophosphatidic Acid Signaling May Initiate Fetal Hydrocephalus. Science Translational Medicine 3, 99ra87, doi: 10.1126/scitranslmed.3002095 (2011). PMC3653407 21900594

[b21] Estivill-Torrus G. . Absence of LPA1 signaling results in defective cortical development. Cereb Cortex 18, 938–950, doi: bhm132 [pii]10.1093/cercor/bhm132 (2008). 17656621 10.1093/cercor/bhm132

[b22] Sumida H. . LPA4 regulates blood and lymphatic vessel formation during mouse embryogenesis. Blood 116, 5060–5070, doi: 10.1182/blood-2010-03-272443 (2010). 20713964

[b23] Contos J. J. A. . Characterization of lpa2 (Edg4) and lpa1/lpa2 (Edg2/Edg4) Lysophosphatidic Acid Receptor Knockout Mice: Signaling Deficits without Obvious Phenotypic Abnormality Attributable to lpa2. Mol. Cell. Biol. 22, 6921–6929, doi: 10.1128/mcb.22.19.6921-6929.2002 (2002). 12215548 PMC134025

[b24] Ye X. . LPA3-mediated lysophosphatidic acid signalling in embryo implantation and spacing. Nature 435, 104–108, doi: http://www.nature.com/nature/journal/v435/n7038/suppinfo/nature03505_S1.html (2005). 15875025 10.1038/nature03505 PMC1369590

[b25] Tigyi G. Aiming drug discovery at lysophosphatidic acid targets. British Journal of Pharmacology 161, 241–270, doi: 10.1111/j.1476-5381.2010.00815.x (2010). 20735414 PMC2989581

[b26] Geach T. J. . An essential role for LPA signalling in telencephalon development. Development (Cambridge, England) 141, 940–949, doi: 10.1242/dev.104901 (2014). 24496630

[b27] Tokumura A. . Identification of Human Plasma Lysophospholipase D, a Lysophosphatidic Acid-producing Enzyme, as Autotaxin, a Multifunctional Phosphodiesterase. Journal of Biological Chemistry 277, 39436–39442, doi: 10.1074/jbc.M205623200 (2002). 12176993

[b28] Umezu-Goto M. . Autotaxin has lysophospholipase D activity leading to tumor cell growth and motility by lysophosphatidic acid production. The Journal of Cell Biology 158, 227–233, doi: 10.1083/jcb.200204026 (2002). 12119361 PMC2173129

[b29] van Meeteren L. A. . Autotaxin, a secreted lysophospholipase D, is essential for blood vessel formation during development. Mol Cell Biol 26, 5015–5022, doi: 26/13/5015 [pii]10.1128/MCB.02419-05 (2006). 16782887 10.1128/MCB.02419-05 PMC1489177

[b30] Tanaka M. . Autotaxin stabilizes blood vessels and is required for embryonic vasculature by producing lysophosphatidic acid. The Journal of biological chemistry 281, 25822–25830, doi: 10.1074/jbc.M605142200 (2006). 16829511

[b31] Lai S.-L. . Autotaxin/Lpar3 signaling regulates Kupffer’s vesicle formation and left-right asymmetry in zebrafish. Development (Cambridge, England) 139, 4439–4448, doi: 10.1242/dev.081745 (2012). 23095890

[b32] Nakanaga K. . Overexpression of autotaxin, a lysophosphatidic acid-producing enzyme, enhances cardia bifida induced by hypo-sphingosine-1-phosphate signaling in zebrafish embryo. Journal of Biochemistry 155, 235–241, doi: 10.1093/jb/mvt114 (2014). 24451492

[b33] Yuelling L., Waggener C., Afshari F., Lister J. & Fuss B. Autotaxin/ENPP2 regulates oligodendrocyte differentiation *in vivo* in the developing zebrafish hindbrain. Glia 60, 1605–1618, doi: 10.1002/glia.22381 (2012). 22821873 PMC3422414

[b34] Yukiura H. . Autotaxin regulates vascular development via multiple lysophosphatidic acid (LPA) receptors in zebrafish. The Journal of biological chemistry 286, 43972–43983, doi: 10.1074/jbc.M111.301093 (2011). 21971049 PMC3243515

[b35] Bisgrove B., Essner J. & Yost H. Multiple pathways in the midline regulate concordant brain, heart and gut left-right asymmetry. Development (Cambridge, England) 127, 3567–3579 (2000). 10.1242/dev.127.16.3567 10903181

[b36] Danos M. & Yost H. Role of notochord in specification of cardiac left-right orientation in zebrafish and Xenopus. Developmental biology 177, 96–103, doi: 10.1006/dbio.1996.0148 (1996). 8660880

[b37] Meno C. . lefty-1 is required for left-right determination as a regulator of lefty-2 and nodal. Cell 94, 287–297, doi: 10.1016/S0092-8674(00)81472-5 (1998). 9708731

[b38] Hirokawa N., Tanaka Y., Okada Y. & Takeda S. Nodal flow and the generation of left-right asymmetry. Cell 125, 33–45 (2006). 16615888 10.1016/j.cell.2006.03.002

[b39] Mercola M. & Levin M. Left-right asymmetry determination in vertebrates. Annual Review of cell and developmental Biology 17, 779–805 (2001). 10.1146/annurev.cellbio.17.1.779 11687504

[b40] Essner J., Amack J., Nyholm M., Harris E. & Yost H. Kupffer’s vesicle is a ciliated organ of asymmetry in the zebrafish embryo that initiates left-right development of the brain, heart and gut. Development (Cambridge, England) 132, 1247–1260, doi: 10.1242/dev.01663 (2005). 15716348

[b41] Borovina A., Superina S., Voskas D. & Ciruna B. Vangl2 directs the posterior tilting and asymmetric localization of motile primary cilia. Nature cell biology 12, 407–412, doi: 10.1038/ncb2042 (2010). 20305649

[b42] Stubbs J., Oishi I., Izpisúa Belmonte J. & Kintner C. The forkhead protein Foxj1 specifies node-like cilia in Xenopus and zebrafish embryos. Nature genetics 40, 1454–1460, doi: 10.1038/ng.267 (2008). 19011629 PMC4648715

[b43] Yu X., Ng C., Habacher H. & Roy S. Foxj1 transcription factors are master regulators of the motile ciliogenic program. Nature genetics 40, 1445–1453, doi: 10.1038/ng.263 (2008). 19011630

[b44] Cooper M. & D’Amico L. A cluster of noninvoluting endocytic cells at the margin of the zebrafish blastoderm marks the site of embryonic shield formation. Developmental biology 180, 184–198, doi: 10.1006/dbio.1996.0294 (1996). 8948584

[b45] Aamar E. & Dawid I. Sox17 and chordin are required for formation of Kupffer’s vesicle and left‐right asymmetry determination in zebrafish. Developmental Dynamics, doi: 10.1002/dvdy.22431 (2010). PMC3090657 20925124

[b46] Schulte-Merker S. . Expression of zebrafish goosecoid and no tail gene products in wild-type and mutant no tail embryos. Development (Cambridge, England) 120, 843–852 (1994). 10.1242/dev.120.4.843 7600961

[b47] Ohta H. . Ki16425, a subtype-selective antagonist for EDG-family lysophosphatidic acid receptors. Mol Pharmacol 64, 994–1005, doi: 10.1124/mol.64.4.994 (2003). 14500756

[b48] Sriwai W., Zhou H. & Murthy K. G(q)-dependent signalling by the lysophosphatidic acid receptor LPA(3) in gastric smooth muscle: reciprocal regulation of MYPT1 phosphorylation by Rho kinase and cAMP-independent PKA. The Biochemical journal 411, 543–551, doi: 10.1042/bj20071299 (2008). 18237278 PMC4872639

[b49] Marlow F., Topczewski J., Sepich D. & Solnica-Krezel L. Zebrafish Rho Kinase 2 Acts Downstream of Wnt11 to Mediate Cell Polarity and Effective Convergence and Extension Movements. Current Biology 12, 876–884 (2002). 12062050 10.1016/s0960-9822(02)00864-3

[b50] Wei L. . Rho kinases play an obligatory role in vertebrate embryonic organogenesis. Development (Cambridge, England) 128, 2953–2962 (2001). 10.1242/dev.128.15.2953 11532918

[b51] Davies S., Reddy H., Caivano M. & Cohen P. Specificity and mechanism of action of some commonly used protein kinase inhibitors. Biochem. J 351, 95–105 (2000). 10998351 10.1042/0264-6021:3510095 PMC1221339

[b52] Spemann H. & Mangold H. Induction of embryonic primordia by implantation of organizers from a different species. 1923. The International journal of developmental biology 45, 13–38 (2000). 11291841

[b53] Shih J. & Fraser S. E. Characterizing the zebrafish organizer: microsurgical analysis at the early-shield stage. Development (Cambridge, England) 122, 1313–1322 (1996). 10.1242/dev.122.4.1313 8620858

[b54] Warga R. & Kimmel C. Cell movements during epiboly and gastrulation in zebrafish. Development (Cambridge, England) (1990). 10.1242/dev.108.4.569 2387236

[b55] Topczewski J. . The zebrafish glypican knypek controls cell polarity during gastrulation movements of convergent extension. Developmental cell 1, 251–264, doi: 10.1016/S1534-5807(01)00005-3 (2001). 11702784

[b56] Wang G. . The Rho kinase Rock2b establishes anteroposterior asymmetry of the ciliated Kupffer’s vesicle in zebrafish. Development (Cambridge, England) 138, 45–54, doi: 10.1242/dev.052985 (2011). PMC2998165 21098560

[b57] Ye D. & Lin F. S1pr2/Galpha13 signaling controls myocardial migration by regulating endoderm convergence. Development (Cambridge, England) 140, 789–799, doi: 10.1242/dev.085340 (2013). PMC3557776 23318642

[b58] Schlessinger K., Hall A. & Tolwinski N. Wnt signaling pathways meet Rho GTPases. Genes & development, doi: 10.1101/gad.1760809 (2009). 19204114

[b59] Winter C. G. . Drosophila Rho-associated kinase (Drok) links Frizzled-mediated planar cell polarity signaling to the actin cytoskeleton. Cell 105, 81–91 (2001). 11301004 10.1016/s0092-8674(01)00298-7

[b60] Zhu S., Liu L., Korzh V., Gong Z. & Low B. RhoA acts downstream of Wnt5 and Wnt11 to regulate convergence and extension movements by involving effectors Rho kinase and Diaphanous: use of zebrafish as …. Cellular signalling (2006). 10.1016/j.cellsig.2005.05.019 16019189

[b61] Wang Y. & Nathans J. Tissue/planar cell polarity in vertebrates: new insights and new questions. Development (Cambridge, England) 134, 647–658, doi: 10.1242/dev.02772 (2007). 17259302

[b62] Kim G.-H. & Han J.-K. JNK and ROKα function in the noncanonical Wnt/RhoA signaling pathway to regulate Xenopus convergent extension movements. Developmental Dynamics 232, 958–968, doi: 10.1002/dvdy.20262 (2005). 15739222

[b63] Moolenaar W. & Perrakis A. Insights into autotaxin: how to produce and present a lipid mediator. Nature reviews. Molecular cell biology 12, 674–679, doi: 10.1038/nrm3188 (2011). 21915140

[b64] Ohkawara B., Yamamoto T. S., Tada M. & Ueno N. Role of glypican 4 in the regulation of convergent extension movements during gastrulation in Xenopus laevis. Development (Cambridge, England) 130, 2129–2138 (2003). 10.1242/dev.00435 12668627

[b65] Koike S. . Autotaxin is required for the cranial neural tube closure and establishment of the midbrain-hindbrain boundary during mouse development. Developmental dynamics: an official publication of the American Association of Anatomists 240, 413–421, doi: 10.1002/dvdy.22543 (2011). 21246658

[b66] Bisgrove B., Morelli S. & Yost H. Genetics of human laterality disorders: insights from vertebrate model systems. *… and human genetics* (2003). 10.1146/annurev.genom.4.070802.110428 12730129

[b67] Compernolle V. . Cardia bifida, defective heart development and abnormal neural crest migration in embryos lacking hypoxia-inducible factor-1α. Cardiovascular Research 60, 569–579, doi: 10.1016/j.cardiores.2003.07.003 (2003). 14659802

[b68] Kimmel C., Ballard W., Kimmel S., Ullmann B. & Schilling T. Stages of embryonic development of the zebrafish. Developmental dynamics: an official publication of the American Association of Anatomists 203, 253–310, doi: 10.1002/aja.1002030302 (1995). 8589427

[b69] Reifers F. . Fgf8 is mutated in zebrafish acerebellar (ace) mutants and is required for maintenance of midbrain-hindbrain boundary development and somitogenesis. Development (Cambridge, England) 125, 2381–2395 (1998). 10.1242/dev.125.13.2381 9609821

[b70] Kaslin J. . Stem cells in the adult zebrafish cerebellum: initiation and maintenance of a novel stem cell niche. The Journal of neuroscience: the official journal of the Society for Neuroscience 29, 6142–6153, doi: 10.1523/JNEUROSCI.0072-09.2009 (2009). 19439592 PMC6665484

