# Supplementary material for: Role of ectonucleotide pyrophosphatase/phosphodiesterase 2 in the midline axis formation of zebrafish
Source: Sci Rep. 2016 Nov 24;6:37678. doi: 10.1038/srep37678 (PMC5121889; doi:10.1038/srep37678)

**Role of ectonucleotide pyrophosphatase/phosphodiesterase 2 in the midline axis formation of zebrafish**

Frisca Frisca^1,2^, Daniel Colquhoun^1^, Yona Goldshmit^1,3^, Minna-Liisa Anko^4^, Alice Pébay^2*^ and Jan Kaslin1*, ^

^1^ *Australian Regenerative Medicine Institute, Level 1, Building 75, Monash University, Australia*

*^2^ Centre for Eye Research Australia, Royal Victorian Eye and Ear Hospital & Ophthalmology, the University of Melbourne, Department of Surgery, Australia.*

*^3^ Department of Neurobiology, Tel-Aviv University, Israel*

*^4^Department of Anatomy and Developmental Biology, Monash University, Australia*

**Supplemental Figure 1.** A. enpp2 overexpression measured by RT-PCR in the early embryogenesis of zebrafish. Developmental series of RT-PCR at designated stages were performed using enpp2 Taqman® probe. Data are mean ± SEM. Statistical analysis was established by one-way ANOVA; * P < 0.05; ** P < 0.01. B. *In situ* hybridisation of *enpp2* at 15 somite stage embryos (ss) using sense probe showing no specific signal in comparison to embryo hybridised with antisense probe (see Figure 1 A).

**Supplemental Figure 2.** Expression pattern of *enpp2* and *lpa_1-3_* by WISH. Developmental series of WISH at designated stages were performed using *enpp2* and *lpa_1-3_* antisense riboprobe. Lateral view and animal/dorsal view of WISH early ZF embryo at designated stages. Scale bars: 200 μM.

**Supplemental Figure 3**. Antagonizing lpa_1-3_ using Ki16425 rescues the enpp2-overexpressed phenotypes. Representative bright field (A) and WISH images with *gli2* riboprobes (B) of rescued experiments using Ki16425; in control (immersed in vehicle), Ki16425 (1 and 5μM) at 24hpf. The phenotype penetrance following enpp2 injection and Ki16425 treatment were measured at 24hpf (C, D). Following enpp2 overexpression, the phenotype observed was 71.1 ± 3.6 % in the vehicle control, 59.5 ± 9.5 % in Ki16425 1 μM, and 29.8 ± 7.5 % in Ki16425 5 μM. (C, D). E-F. Rescue experiments in *enpp2* overexpressing embryos with Ki16425and Y27632 using *ntl* and *spaw* WISH as readout at 24 hpf. Following enpp2 overexpression, the *ntl* phenotype (notochord defects) was 70 ± 7.1 % in the vehicle control, 30± 8.5 % in Ki16425 7.5 μM, and 26 ± 4.3 % in Y27632 10 μM. *spaw* is normally expressed on the left side of the midline. The *spaw* expression phenotype (Figure 2 C, no expression, bilateral expression or expression on right side of the midline) was rescued in 36 ± 4.3 % of the Y27632 treated enpp2 overexpressing embryos. There was no significant rescue of *spaw* expression in Ki16425 treated embryos, although a clear trend was detected. Total number of embryos analysed stated above bar. Total number of embryos pooled from two independent experiments. Data are mean ± SEM. Statistical analysis was established by one-way ANOVA; * P < 0.05; ** P < 0.01. Scale bars: 200 μM.

**Supplemental Movie 1-2**. Representative images of time lapse series showing cell migration during midline formation in wild type and enpp2-injected embryo. Image stacks were taken every 20 minutes for 9 hours. The enpp2 overexpressed embryo in failed to undergo a proper CE which results in bend and broadened midline compared to wild type H2B-GFP labelled cells in green.

**Suppl. Figure 1**


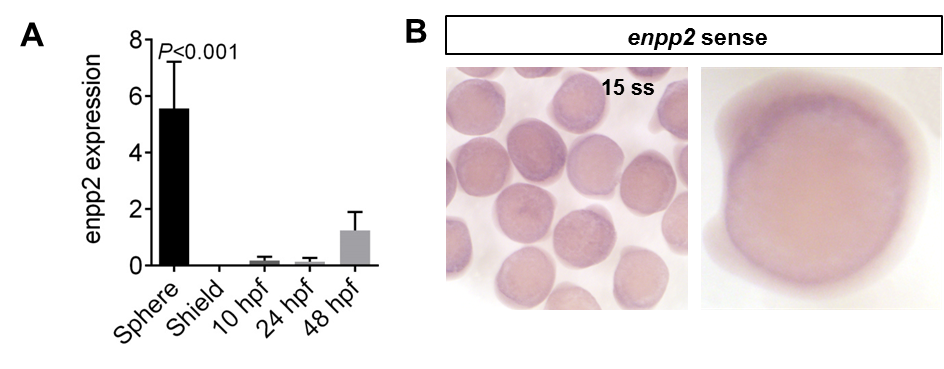


**Suppl. Figure 2**


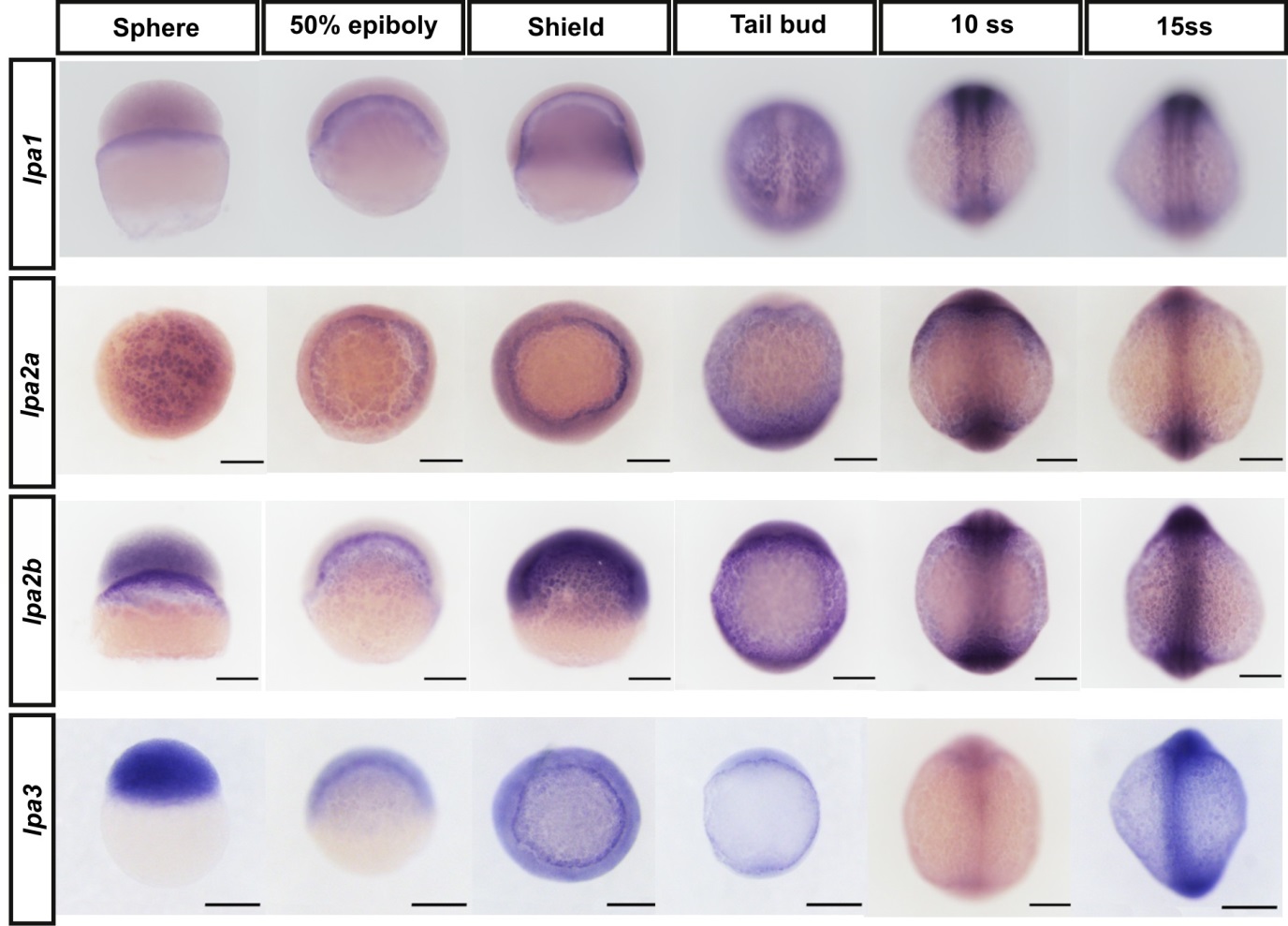


**Suppl. Figure 3**


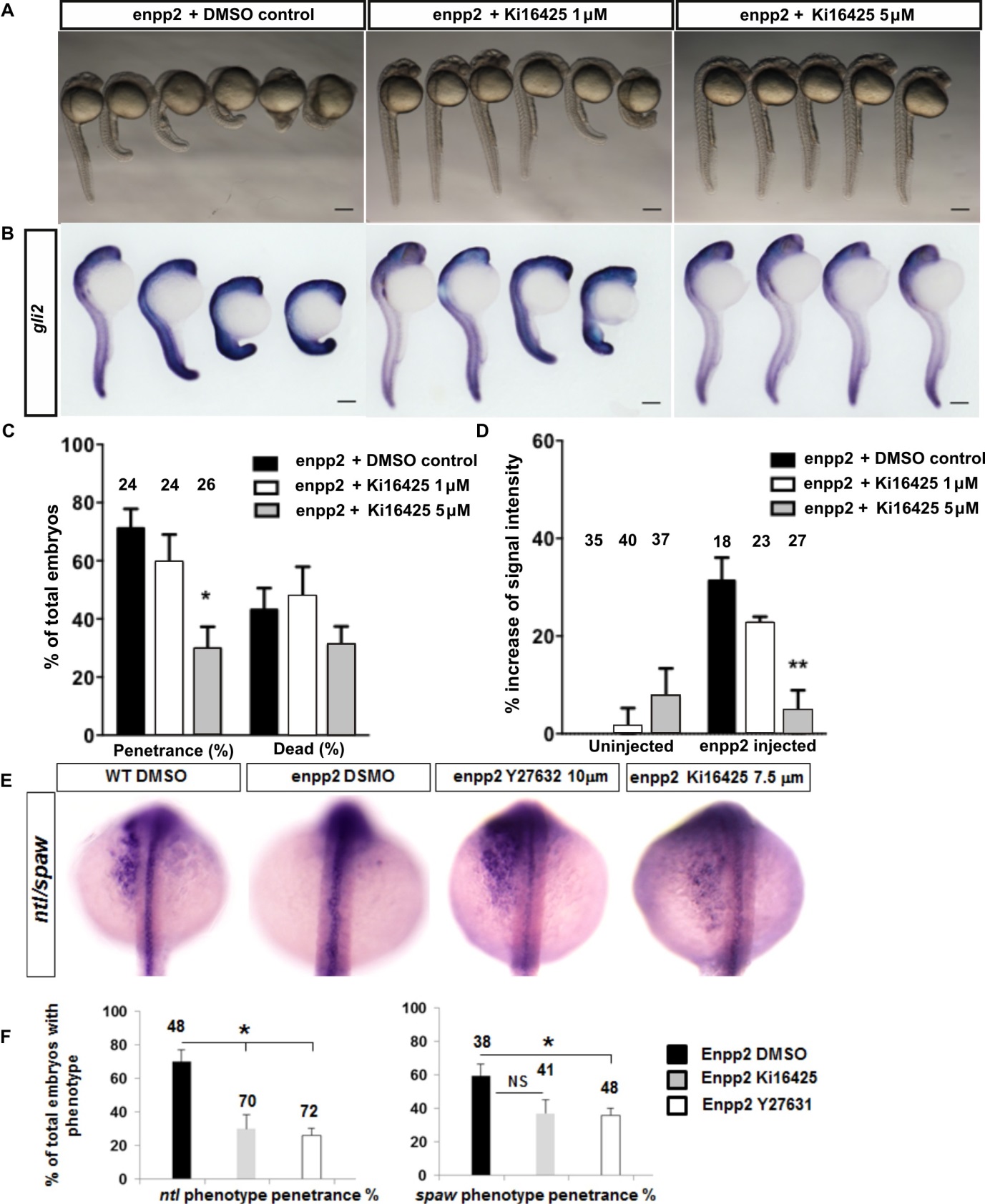

Supplement: Supplementary Figures 1–3 [file srep37678-s1.doc]
